# Artesunate-amodiaquine fixed dose combination for the treatment of *Plasmodium falciparum *malaria in India

**DOI:** 10.1186/1475-2875-11-97

**Published:** 2012-03-30

**Authors:** Anupkumar R Anvikar, Bhawna Sharma, Bhartendu H Shahi, Prajesh K Tyagi, Tarit K Bose, Surya K Sharma, Prakriti Srivastava, Bina Srivastava, Jean R Kiechel, Aditya P Dash, Neena Valecha

**Affiliations:** 1 National Institute of Malaria Research, Sector 8, Dwarka, New Delhi 110077, India; 2 Drugs for Neglected Diseases initiative (DNDi), Tuberculosis Association of India Building, 1st Floor, 3 Red Cross Road, Near Parliament House, New Delhi, India; 3 National Institute of Malaria Research, IDVC Field Unit, Sector 5, Rourkela, Odisha, India; 4 Community Welfare Society Hospital Jagda, Rourkela, Odisha, India; 5 Drugs for Neglected Diseases initiative (DNDi), 15 Chemin Louis-Dunant, 1202 Geneva, Switzerland

**Keywords:** Artesunate, Amodiaquine, *falciparum *malaria, India

## Abstract

**Background:**

Artemisinin-based combination therapy (ACT) has been recommended for the treatment of *falciparum *malaria by the World Health Organization. Though India has already switched to ACT for treating *falciparum *malaria, there is need to have multiple options of alternative forms of ACT. A randomized trial was conducted to assess the safety and efficacy of the fixed dose combination of artesunate-amodiaquine (ASAQ) and amodiaquine (AQ) for the treatment of uncomplicated *falciparum *malaria for the first time in India. The study sites are located in malaria-endemic, chloroquine-resistant areas.

**Methods:**

This was an open label, randomized trial conducted at two sites in India from January 2007 to January 2008. Patients between six months and 60 years of age having *Plasmodium falciparum *mono-infection were randomly allocated to ASAQ and AQ arms. The primary endpoint was 28-day PCR-corrected parasitological cure rate.

**Results:**

Three hundred patients were enrolled at two participating centres, Ranchi, Jharkhand and Rourkela, Odisha. Two patients in AQ arm had early treatment failure while there was no early treatment failure in ASAQ arm. Late treatment failures were seen in 13 and 12 patients in ASAQ and AQ arms, respectively. The PCR-corrected cure rates in intent-to-treat population were 97.51% (94.6-99.1%) in ASAQ and 88.65% (81.3-93.9%) in AQ arms. In per-protocol population, they were 97.47% (94.2-99.2%) and 88.30% (80-94%) in ASAQ and AQ arms respectively. Seven serious adverse events (SAEs) were reported in five patients, of which two were reported as related to the treatment. All SAEs resolved without sequel.

**Conclusion:**

The fixed dose combination of ASAQ was found to be efficacious and safe treatment for *P. falciparum *malaria. Amodiaquine also showed acceptable efficacy, making it a suitable partner of artesunate. The combination could prove to be a viable option in case India opts for fixed dose combination ACT.

**Clinical trial registry:**

ISRCTN84408319

## Background

The World Health Organization (WHO) has recommended artemisinin-based combination therapy (ACT) as the treatment for *Plasmodium falciparum *and many malaria endemic countries are using it. The combination of amodiaquine and artesunate, along with four more combinations, is recommended by WHO for malaria control programmes [1].

India has switched over to ACT and the National Vector Borne Disease Control Programme recommends the use of artesunate + sulphadoxine-pyrimethamine (AS + SP) for the treatment for *falciparum *malaria [2]. There is not much data available on the efficacy of the partner drug SP, but there is evidence of *dhfr *and *dhps *mutations [3] and treatment failure has been reported in north-eastern states [4]. Further, since the combination is available as blister pack, compliance may be poor and this provides opportunity for consuming monotherapy. There is also the issue of dosage in paediatric age group. This forms the basis of evaluation of different forms of ACT, which may form an alternative to AS + SP combination.

The combination artesunate-amodiaquine (ASAQ) has been extensively studied and good efficacy and tolerability has been reported. A systematic review of relevant studies [5-8] on the treatment of uncomplicated *P*. *falciparum *malaria conducted over the past 10 years in Africa showed that amodiaquine (AQ) proved significantly more effective than chloroquine in clearing parasites, with a tendency for faster clinical recovery. This difference was also observed in areas with considerable chloroquine resistance. Further, serious adverse events have not been reported with curative short-term regimen of AQ [9].

A randomized trial was conducted to assess the safety and efficacy of the fixed dose combination of ASAQ and AQ alone for treatment of uncomplicated *falciparum *malaria for the first time in India. The study sites were located in malaria-endemic, chloroquine-resistant areas.

## Methods

**Figure 1:**
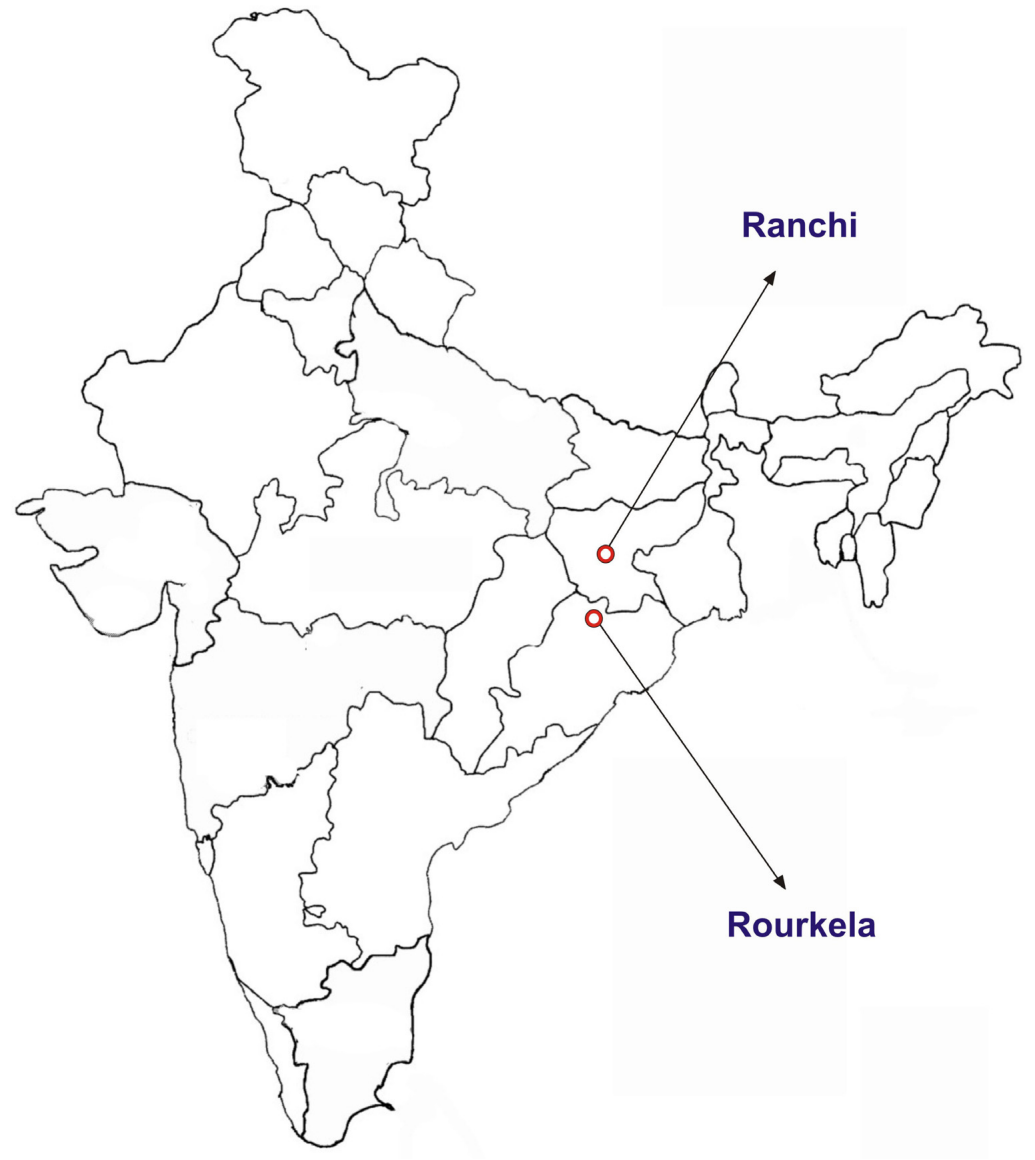
**Map of India showing the study sites**.

This was an open label, randomized study carried out at Mahadevi Birla Hospital, Ranchi, Jharkhand and Community Welfare Society Hospital, Rourkela, Odisha from January 2007 to January 2008 (Figure 1). Rourkela is situated in a high malaria-endemic district, Sundargarh where malaria transmission is perennial with proportion of *P. falciparum *cases of about 96%. Ranchi district has peak malaria season from August to November with relatively low proportion (50%) of *falciparum *cases. The efficacy of chloroquine was 54 to 57% in Sundargarh and 72% in Ranchi in an earlier study [10]. AS + SP was the recommended combination for *falciparum *malaria in both the study areas during this period.

### Patients

The study was carried out in patients aged from six months to 60 years, weighing more than 5 kg and having *P. falciparum *mono-infection (asexual parasitaemia of 1,000-100,000 per μl) and fever ≥37.5°C (99.5°F). Patients with signs of severe malaria, febrile conditions due to diseases other than malaria, known severe disease, history of hypersensitivity to drug(s), positive pregnancy test or lactating women, history of anti-malarial treatment in past 15 days were excluded. Patients were also excluded from the study if they had anaemia (haemoglobin < 7 g/dl), hepatic (alanine aminotransferase/aspartate aminotransferase ≥ 2.5 ULN) or renal impairment (serum creatinine ≥ 1.2 ULN).

### Randomization and treatment

Patients were randomized to one of the two arms in 2:1 ratio; fixed dose combination tablets of ASAQ and AQ tablets alone using a predetermined randomization list. Individual, opaque, sealed and sequentially numbered envelopes were used for randomization. ASAQ was given once daily orally on days 0, 1 and 2 of the study according to age group. Two different strengths of the combination were formulated; paediatric/lower strength (AS: 25 mg/AQ: 67.5 mg) and adult/higher strength (AS: 100 mg/AQ: 270 mg). Children in the six to 11 months age group were given one lower strength tablet, one to five years, two lower strength tablets, six to 13 years one higher strength tablet and those older than 14 years were given two higher strength tablets. AQ tablets (153 mg base) were administered orally, once daily for three days. The number of tablets was decided according to age and weight with the adult dose being four tablets on day 0, 1 and three tablets on day 2.

### Safety and efficacy assessments

Anti-malarial drug efficacy was assessed by using the WHO protocol with a 28-day follow up [11]. Participating patients were hospitalized for a period of three days and later followed on days 7, 14, 21 and 28 of enrolment. They were also asked to make an unscheduled visit on any day on which they felt unwell.

The primary endpoint of the study was the PCR -corrected cure rate based on the adequate clinical and parasitological response, which is defined as the absence of parasitaemia, irrespective of patient's body temperature, with the patient not meeting any criteria of early treatment failure or late clinical or parasitological failure as defined by the WHO [11].

The secondary endpoints included the Parasite Reduction Ratio, Parasite Clearance Time, Fever Clearance Time, percent patients without gametocytes on day 28 and proportion of patients with early and late treatment failure and late parasitological failure.

### Laboratory analyses

Blood samples were collected by fingerpick method for parasitological assessment at enrolment, during admission and follow up visits. Thick and thin smears were prepared, Giemsa-stained and examined for parasite density. This was done by counting the number of asexual parasites per 200 leucocytes in thick blood film. Parasite density per μl was calculated as (number of parasites counted × 8,000)/(number of leukocytes counted). Two qualified microscopists independently read all of the slides and parasite densities were calculated by averaging the two counts. Two to three drops of blood were collected on a chromatography filter paper no.3 (Whatman, UK) from each patient at inclusion. A second specimen was collected only in the case of reappearance of parasites as evidenced by a positive microscopy slide. Genotyping was carried out at NIMR, Delhi. For differentiating recrudescence from re-infection, three genetic markers, merozoite surface proteins (*msp1 *and *msp2*) and glutamate rich protein (*glurp*) were used [12].

Blood samples were drawn for haematologic (haemoglobin level, leucocyte count, haematocrit) and biochemical (serum alanine aminotransferase, aspartate aminotransferase, bilirubin and creatinine) assessments on day 0, 7, 28 and on the day of recurrent parasitaemia.

### Statistical analysis

This was not a head to head comparative study but rather a parallel study in which the AQ alone arm acted as a positive control. Hence, the sample size was estimated using the precision method with a two-sided alpha of 0.05 and a power of 0.8. On the assumption that ASAQ will have a cure rate of 95% and a precision of 5%, the estimated sample size was 73. If a cure rate of 90% was considered with precision of 5%, the sample size increased to 138. Hence, it was planned to recruit about 300 patients; 200 in ASAQ and 100 in AQ arm. Epi Info 6.04b software was used for these calculations.

Three patient populations were evaluated. The safety population comprised all patients who were randomized and received at least one dose of study drug. The safety analysis and listing was done on safety population. The intent to treat (ITT) population comprised of all patients who were randomized, received at least one dose of study drug and underwent at least one efficacy assessment. The secondary efficacy analysis was performed on ITT population. Per protocol (PP) population comprised of all patients who completed the study as per the protocol. The primary and secondary efficacy analysis was performed on PP population. Patients who withdrew from the study or did not appear for the scheduled visits on or before day 28 were considered as drop-outs. These patients were part of ITT and safety population and hence their data were reported.

The primary endpoint of day 28 cure rate was evaluated in both ITT and PP populations. Cure rates were based on simple proportions. The 95% confidence interval was calculated for both the treatment arms. The secondary efficacy parameter parasite reduction ratio at 48 hr and Parasite and Fever clearance times were analyzed in ITT population using Kaplan Meier analysis. Statistical analyses were done using the software SAS 9.1.3.

### Ethical issues

The study was conducted in accordance with the local laws and regulations including the schedule Y, Indian Good Clinical Practices, Ethical guidelines on biomedical research issued by the Indian Council of Medical Research, International Conference on Harmonization - Good Clinical Practice (ICH-GCP). The protocol was reviewed and approved by the Ethics Committees of the National Institute of Malaria Research, Delhi and Community Welfare Society Hospital, Rourkela. Written informed consent was obtained from participants/guardians. In case of an illiterate patient, his/her thumb impression and signature of an independent witness was obtained.

## Results

**Figure 2:**
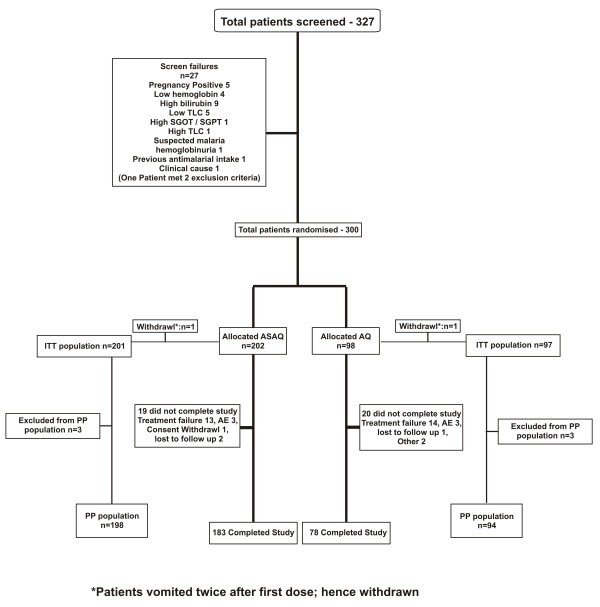
**Participant flow**.

Three hundred and twenty seven patients were screened at the two sites of which, 300 were randomized to the two treatment arms. The ITT population comprised of 298 subjects, 201 in ASAQ and 97 in AQ arm (Figure 2). The demographic parameters of the patients enrolled in both the arms are shown as Table 1 and revealed no significant difference.

**Table 1:** Demographic parameters of patients enrolled in the study

Parameters	ASAQ arm (N = 202)	AQ arm (N = 98)
Gender		

*Male*	118	67

*Female*	84	31

Age (years)		

*< 1 year*	1	0

*1-12 years*	70	26

*> 12 years*	131	72

Mean Weight (kg)	38.30	42.94

Mean Parasite density (/μl)	26650	29270

Mean(± SD) axillary temperature	100.7 ± 0.91	100.6 ± 0.92

In ITT population, the 28-day PCR uncorrected cure rates were 91.54% (86.8%-95%) and 81.44% (72.3%-88.6%) in ASAQ and AQ arms respectively. In PP population, they were 92.42% (87.8-95.7%) in ASAQ arm and 82.98% (72.8-89.9%) in AQ arm (Table 2).

**Table 2:** Cure rates in ITT and PP populations

	Ranchi	Rourkela	Overall
	**Cure rate (95% C.I.)**	**Cure rate (95% C.I.)**	**Cure rate (95% C.I.)**

***ITT population***			

*PCR uncorrected*			

ASAQ	88.57 (80.9 - 94.0)	94.79 (88.3 - 98.3)	91.54 (86.8 - 95.0)

AQ	88.00 (75.7 - 95.5)	74.47 (59.7 - 86.1)	81.44 (72.3 - 88.6)

*PCR corrected*			

ASAQ	97.14 (91.9 - 99.4)	97.92 (92.7 - 99.7)	97.51 (94.6 - 99.1)

AQ	96.00 (86.3 - 99.5)	80.85 (66.7 - 90.9)	88.65 (81.3 - 93.9)

***PP population***			

PCR uncorrected			

ASAQ	89.42 (81.9 - 94.6)	95.74 (89.5 - 98.8)	92.42 (87.8 - 95.7)

AQ	89.58 (77.3 - 96.5)	76.09 (61.2 - 87.4)	82.99 (73.8 - 89.9)

PCR corrected			

ASAQ	97.12 (91.8 - 99.4)	97.87 (92.5 - 99.7)	97.47 (94.2 - 99.2)

AQ	95.83 (86.7 - 99.5)	80.43 (66.1 - 90.6)	88.30 (80.0 - 94.0)

There were 27 treatment failures (13 in ASAQ and 14 in AQ arm). The PCR analysis showed that six subjects had new infections (five in ASAQ and one in AQ arm) and 16 had recrudescence (five in ASAQ and 11 in AQ arm). The mean age of failure patients was 21.8 and 13.2 years in ASAQ and AQ arms respectively. The mean initial parasitaemia was 49065 and 37862 respectively in ASAQ and AQ arms. Four samples were inconclusive (three in ASAQ and one in AQ arm), while result could not be obtained in one sample (AQ arm). The patients with indeterminate PCR results were classified as recrudescent infections whereas the new infections were classified as 'cured'.

After PCR correction, the cure rates were 97.51% (94.6-99.1%) and 88.65% (81.3-93.9%) in ITT population. PCR corrected cure rates in PP population were 97.47% (94.2-99.2%) in ASAQ and 88.30% (95% CI 80.0-94.0%) in AQ arm (Table 2). Site wise cure rates in both populations have been shown in Table 2. Cure rates were similar in ASAQ at both the sites [97.12% (91.8-99.4%) at Ranchi and 97.87% (92.5-99.7%) at Rourkela]. Cure rate of AQ was higher in Ranchi [95.83% (86.7-99.5%)] as compared to Rourkela [80.43% (66.1-90.6%)] but the difference was not statistically significant (p > 0.05).

### Parasite clearance and resolution of clinical symptoms

The mean parasite densities at enrolment were similar in both the treatment arms. The median parasite clearance time was one day in ASAQ arm, while it was two days in AQ arm showing that parasite clearance was significantly faster in ASAQ arm (p < 0.0001). The mean parasite clearance time was 1.5 and 2.1 days in ASAQ and AQ arms respectively.

**Figure 3:**
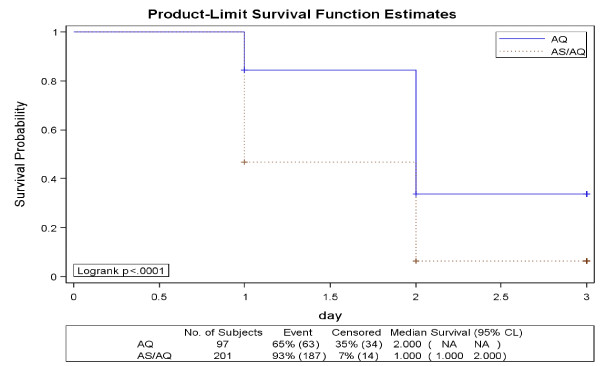
**Survival curve (Kaplan Meier plots) of Parasite Clearance Time (ITT population)**.

The Kaplan Meier estimate of the proportion of aparasitaemic patients at 48 hours was 93% (187/201) in ASAQ arm and 65% (63/97) in AQ arm (Figure 3). The number of patients completing treatment in ASAQ and AQ arms was 200 and 94 respectively. Three patients were withdrawn from the study before 48 hours (one adverse event in ASAQ and two early treatment failures in AQ arm).

**Figure 4:**
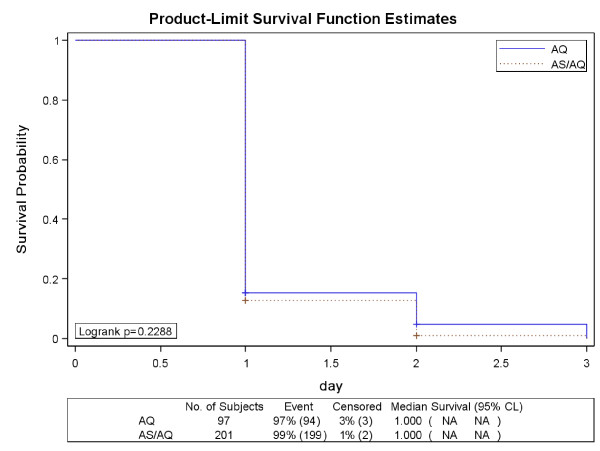
**Survival curve (Kaplan Meier plots) of Fever Clearance Time (ITT population)**.

The Parasite Reduction Ratio was 1,107.5 in ASAQ arm and 138.8 in AQ arm; the difference was significant (p = 0.002). The median Fever Clearance Time was same (one day) in both the treatment arms (Figure 4).

There was no early treatment failure in ASAQ arm while two (2.1%) patients had early treatment failure in AQ arm. The proportion of patients with late treatment failures was 6.5% (13/201) in ASAQ and 12.4% (12/97) in AQ arms. The proportion of patients with gametocytes at the end of follow-up (day 28) was 1.5% (03/201) in ASAQ and 2.1% (02/97) in AQ arms. The late clinical failures, which represented patients from day 4 to 28 with parasitaemia and fever were 4.0% (8/201) in ASAQ and 5.2% (5/97) in AQ arms. The late parasitological failures defined as presence of parasitaemia from day 7 to day 28 were 2.5% (05/201) in ASAQ and 7.2% (07/97) in AQ arms. None of these parameters was statistically significant in the two treatment arms (p > 0.05).

### Protocol deviations

There were in total 10 protocol deviations and five protocol violations in the study. Of the 10 protocol deviations, nine were related to the study procedures. One protocol violation was related to the study assessment when the patient was not available at the correct time point.

### Drug safety

The adverse event (AE) profiles for ASAQ and AQ treatments were similar in terms of type and frequency of events and were mostly those expected in malaria patients. More than half the patients (57.42% in ASAQ and 59.18% in AQ) experienced AE (Table 3). The difference between the incidence of AEs in the two treatment arms was not statistically significant (Chi square = 0.084, p > 0.05). The AEs related to the study medication were six (3.0%) in ASAQ and four (4.1%) in AQ arms respectively. The most common unrelated AEs encountered during the study were the manifestations of the disease: fever, body pain, fatigue and asthenia during the hospitalized observation period. The more common AEs related to the medication in both the arms were the gastrointestinal symptoms: nausea, vomiting and abdominal pain. Their occurrence was 2.5% and 3.1% in ASAQ and AQ arms respectively. There were four cases of vomiting (two each in ASAQ and AQ arms) which were reported as AEs, assessed by the investigator as related to the study medication and required medical intervention. They were resolved within a day following treatment.

**Table 3:** Adverse events by treatment groups (safety population)

System organ class	ASAQ arm (N = 202) n (%)	AQ arm (N = 98) n (%)
**Total subjects with at least one AE**	114 (56.4)	58 (59.2)

Blood and lymphatic system disorders	23 (11.4)	7 (7.1)

Eye disorders	1 (0.5)	0 (0.0)

Gastrointestinal disorders	56 (27.7)	28 (28.6)

General disorders	53 (26.2)	35 (35.7)

Hepato-biliary disorders	2 (1.0)	2 (2.0)

Immune System disorders	1 (0.5)	0 (0.0)

Infections and Infestations	1 (0.5)	0 (0.0)

Investigation abnormalities	13 (6.4)	3 (3.1)

Metabolism and nutrition disorders	0 (0.0)	1 (1.0)

Musculoskeletal and connective tissue disorders	2 (1.0)	5 (5.1)

Nervous system disorders	18 (8.9)	14 (14.3)

Respiratory, thoracic and mediastinal disorders	5 (2.5)	2 (2.0)

Skin and subcutaneous tissue disorders	0 (0.0)	1 (1.0)

Vascular disorders	3 (1.5)	2 (2.0)

Serious adverse events (SAEs) were reported in five patients, three (1.5%) in ASAQ and two (2%) in AQ arm. They were leukocytosis, diarrhoea, vomiting, hyperbilirubinaemia and hypersensitivity reaction. In the ASAQ arm, two SAEs were considered drug related by investigators. One was angioedema without signs of anaphylaxis and another, severe diarrhoea requiring hospitalization. The SAEs in the AQ arm were unrelated to the drug. All SAEs resolved without sequelae.

The haematological and biochemical values on day 0 are shown in Table 4. The mean haemoglobin value was 9.8 ± 1.8 g/dL and 10.1 ± 1.9 g/dL in ASAQ and AQ arms respectively. There was a marginal increase in the level over 28 day period in both the arms.

**Table 4:** Biochemical characteristics of Patients on Day 0, 7, and 28

	ASAQ			AQ		
	
	D0	D7	D28	D0	D7	D28
Haemoglobin (g/dL)	9.8 ± 1.8	10 ± 1.52	11 ± 1.41	10.1 ± 1.94	10.4 ± 1.94	11.2 ± 1.51

RBC (million/mm^3^)	4.6 ± 0.67	4.6 ± 0.62	5.1 ± 0.6	4.7 ± 0.71	4.8 ± 0.66	5.1 ± 0.65

WBC (/mm^3^)	6851 ± 2043.49	7776.4 ± 1644.18	7384.2 ± 1382.73	6934.7 ± 1791.57	7537.6 ± 1678.31	7444.6 ± 1203.77

Total Bilirubin (iu/L)	1.1 ± 0.63	0.6 ± 0.24	0.5 ± 0.23	1.2 ± 0.67	0.6 ± 0.25	0.5 ± 0.29

SGOT (iu/L)	32.2 ± 11.34	28.3 ± 8.48	24.8 ± 10.73	31.4 ± 9.18	26.8 ± 5.67	24.4 ± 7

SGPT (iu/L)	26 ± 11.23	25.2 ± 13.59	19.5 ± 8.29	24.8 ± 10.92	23.3 ± 8.81	20.1 ± 9.39

Creatinine (iu/L)	0.9 ± 0.16	0.8 ± 0.14	0.8 ± 0.11	0.9 ± 0.16	0.9 ± 0.16	0.8 ± 0.13

There were no significant changes in RBC and WBC counts over 28 days. There was a marginal decrease in the levels of total bilirubin, SGOT, SGPT and creatinine over this period.

## Discussion

The study shows that ASAQ fixed dose combination is efficacious for treatment of *falciparum *malaria in India. The PCR-corrected cure rates were 97.47% (95% CI 94.2 - 99.2%) and 88.3% (95% CI 80.0 - 94.0%) in ASAQ and AQ arms respectively. The cure rates were similar in different age groups. The study also supports the role of AQ as a suitable partner in ASAQ. The cure rates in PP population were similar to that in ITT population. Further, they were similar at both the sites. The combination has the advantage of once daily administration, thus increasing compliance. The results of this study are in line with the results obtained in Cameroon, Madagascar, Mali [13], Senegal [13,14], and Ivory Coast [14] and Myanmar [15]. A recent study carried out in Senegal has shown the utility of ASAQ in recurrent uncomplicated falciparum malaria [16]

Currently, AS + SP has been recommended by the National Vector Borne Disease Control Programme in India. The combination is efficacious [17] but treatment failures have been reported with the partner drug SP [4]. There is also evidence of *dhfr *and *dhps *mutations especially in the north-east [3]. Further, since the combination is available as blister pack, compliance may be poor and also provides opportunity of consuming artesunate mono-therapy. Hence, there is need to shift to fixed dose ACT. ASAQ fixed dose combination could be one of the options since it is safe, efficacious and the partner drug AQ also has an acceptable efficacy at least in the study areas. Amodiaquine is known to have cross-resistance with chloroquine. However, studies have shown ASAQ to be effective even in areas with chloroquine resistance [18]. Even the present study was carried out in areas with chloroquine resistance. The utility of ASAQ has also been demonstrated in home management of malaria [19] and also as intermittent preventive therapy in children [20]. The combination can also be useful for vivax malaria and clinically suspected malaria.

According to WHO, to qualify as ACT the combination should also have independent anti-malarial activity [1]. The study showed that AQ had PCR-corrected efficacy of 88.3% (80.0 - 90.6%). The combination is also one of the five WHO prequalified forms of ACT.

Fever was the most common AE but was considered unrelated to the study medication and caused by malaria itself. In the related subgroup, nausea and vomiting were the more common AEs, and they may indicate the gastric irritation caused by the study medication.

The drug has been registered for marketing in India on the basis of results of this study.

## Conclusion

Fixed dose combination ASAQ proved to be an efficacious and safe treatment for *falciparum *malaria in both the study areas. The study also showed that the partner drug, AQ was effective in the study areas, making it a suitable partner of artesunate. The combination could prove to be one of the viable options in case India opts for fixed-dose combination ACT.

## Abbreviations

ACT: Artemisinin based combination therapy; AQ: Amodiaquine; ASAQ: Artesunate amodiaquine; AS + SP: Artesunate + sulphadoxine-pyrimethamine; ITT: Intent to treat; PCR: Polymerase chain reaction; PP: Per protocol; SAE: Serious adverse event

## Competing interests

The authors declare that they have no competing interests.

## Authors' contributions

NV, JK and BS conceived the idea. NV was the PI and study coordinator. NV and BS also supervised all the field sites. AD was involved in overall supervision. AA, BHS, TKB, PKT, SS were involved in field work. PS did the molecular analysis. BS2 was involved in quality assurance of microscopy. All authors read and approved the manuscript.

## References

[B1] World Health Organization Guidelines for the treatment of malaria. Second Edition 2010 WHO, Geneva, Switzerland http://www.who.int/malaria/publications/atoz/9789241547925/en/index.html

[B2] National Vector Borne Disease Control Programme National drug policy on malaria 2010 Directorate of Health Services, Government of India http://nvbdcp.gov.in/Doc/drug-policy-2010.pdf

[B3] Ahmed A Bararia D Vinayak S Yameen M Biswas S Dev V Kumar A Ansari AM Sharma YD *Plasmodium falciparum *isolates in India exhibit a progressive increase in mutations associated with sulfadoxine-pyrimethamine resistance Antimicrob Agents Chemother 2004 48 879 889 10.1128/AAC.48.3.879-889.2004 14982779 PMC353157

[B4] Shah NK Dhillon GP Dash AP Arora U Meshnick SR Valecha N Antimalarial drug resistance of *Plasmodium falciparum *in India: changes over time and space Lancet Infect Dis 2011 11 57 64 10.1016/S1473-3099(10)70214-0 21183147 PMC3068018

[B5] Adjuik M Agnamey P Babiker A Borrmann S Brasseur P Cisse M Cobelens F Diallo S Faucher JF Garner P Gikunda S Kremsner PG Krishna S Lell B Loolpapit M Matsiegui PB Missinou MA Mwanza J Ntoumi F Olliaro P Osimbo P Rezbach P Some E Taylor WR Amodiaquine-artesunate versus amodiaquine for uncomplicated *Plasmodium falciparum *malaria in African children: a randomised multicentre trial Lancet 2002 359 1365 1372 10.1016/S0140-6736(02)08348-4 11978332

[B6] Koram KA Abuaku B Duah N Quashie N Comparative efficacy of antimalarial drugs including ACTs in the treatment of uncomplicated malaria among children under 5 years in Ghana Acta Trop 2005 95 194 203 10.1016/j.actatropica.2005.06.018 16054584

[B7] Hamour S Melaku Y Keus K Wambugu J Atkin S Montgomery J Ford N Hook C Checchi F Malaria in the Nuba Mountains of Sudan: baseline genotypic resistance and efficacy of the artesunate plus sulfadoxine-pyrimethamine and artesunate plus amodiaquine combinations Trans R Soc Trop Med Hyg 2005 99 548 554 10.1016/j.trstmh.2004.10.003 15869770

[B8] Barennes H Nagot N Valea I Koussoubé-Balima T Ouedraogo A Sanou T Yé S A randomized trial of amodiaquine and artesunate alone and in combination for the treatment of uncomplicated *falciparum *malaria in children from Burkina Faso Trop Med Int Health 2004 9 438 444 10.1111/j.1365-3156.2004.01224.x 15078261

[B9] Olliaro P Nevill C LeBras J Ringwald P Mussano P Garner P Brasseur P Systematic review of amodiaquine treatment in uncomplicated malaria Lancet 1996 2 1196 1201 8898036 10.1016/S0140-6736(96)06217-4

[B10] Valecha N Joshi H Mallick PK Sharma SK Kumar A Tyagi PK Shahi B Das MK Nagpal BN Dash AP Low efficacy of chloroquine: time to switchover to artemisinin-based combination therapy for falciparum malaria in India Acta Trop 2009 111 21 28 10.1016/j.actatropica.2009.01.013 19426658

[B11] World Health Organization Assessment and monitoring of anti-malarial drug efficacy for the treatment of uncomplicated falciparum malaria Geneva 2003 http://www.emro.who.int/rbm/publications/protocolwho.pdf

[B12] Snounou G Zhu X Siripoon N Jarra W Thaithong S Brown KN Viriyakosol S Biased distribution of msp1 and msp2 allelic variants in *Plasmodium falciparum *populations in Thailand Trans R Soc Trop Med Hyg 1999 93 369 374 10.1016/S0035-9203(99)90120-7 10674079

[B13] Ndiaye JL Randrianarivelojosia M Sagara I Brasseur P Ndiaye I Randrianasolo L Ratsimbasoa A Forlemu D Moor VA Traore M Dicko Y Dara N Lameyre V Diallo M Djimde A Ekobo AS Gaye O Randomized, multicentre assessment of the efficacy and safety of ASAQ - a fixed-dose artesunate-amodiaquine combination therapy in the treatment of uncomplicated *Plasmodium falciparum *malaria Malar J 2009 8 125 10.1186/1475-2875-8-125 19505304 PMC2698916

[B14] Faye B Offianan AT Ndiaye JL Tine RC Touré W Djoman K Sylla K Ndiaye PS Penali L Gaye O Efficacy and tolerability of artesunate-amodiaquine (Camoquin plus) versus artemether-lumefantrine (Coartem) against uncomplicated *Plasmodium falciparum *malaria: multisite trial in Senegal and Ivory Coast Trop Med Int Health 2010 15 608 613 20214761 10.1111/j.1365-3156.2010.02487.x

[B15] Smithuis F Kyaw MK Phe O Win T Aung PP Oo AP Naing AL Nyo MY Myint NZ Imwong M Ashley E Lee SJ White NJ Effectiveness of five artemisinin combination regimens with or without primaquine in uncomplicated falciparum malaria: an open-label randomised trial Lancet Infect Dis 2010 10 673 681 10.1016/S1473-3099(10)70187-0 20832366 PMC2947715

[B16] Ndiaye JL Faye B Gueye A Tine R Ndiaye D Tchania C Ndiaye I Barry A Cissé B Lameyre V Gaye O Repeated treatment of recurrent uncomplicated *Plasmodium falciparum *malaria in Senegal with fixed-dose artesunate plus amodiaquine versus fixed-dose artemether plus lumefantrine: a randomized, open-label trial Malar J 2011 10 237 10.1186/1475-2875-10-237 21838909 PMC3171378

[B17] National Institute of Malaria Research Annual Report 2010-11 NIMR, Delhi http://www.mrcindia.org/annual-rep/2010-11.pdf

[B18] Taylor WR Rigal J Olliaro PL Drug resistant falciparum malaria and the use of artesunate-based combinations: focus on clinical trials sponsored by TDR J Vector Borne Dis 2003 40 65 72 15119074

[B19] Ratsimbasoa A Ravony H Vonimpaisomihanta JA Raherinjafy R Jahevitra M Rapelanoro R Rakotomanga Jde D Malvy D Millet P Ménard D Compliance, safety, and effectiveness of fixed-dose artesunate-amodiaquine for presumptive treatment of non-severe malaria in the context of home management of malaria in Madagascar Am J Trop Med Hyg 2012 86 203 210 10.4269/ajtmh.2012.11-0047 22302849 PMC3269268

[B20] Ahorlu CK Koram KA Seake-Kwawu A Weiss MG Two-year evaluation of Intermittent Preventive Treatment for Children (IPTc) combined with timely home treatment for malaria control in Ghana Malar J 2011 10 127 10.1186/1475-2875-10-127 21569634 PMC3117750

